# Differential Trajectories of Fathers’ Postpartum Depressed Mood: A Latent Class Growth Analysis Approach

**DOI:** 10.3390/ijerph19031891

**Published:** 2022-02-08

**Authors:** Hsi-Ping Nieh, Chien-Ju Chang, Li-Tuan Chou

**Affiliations:** Department of Human Development and Family Studies, National Taiwan Normal University, 162, Heping East Road, Section 1, Taipei 106, Taiwan; hsipingnieh@ntnu.edu.tw (H.-P.N.); t10011@ntnu.edu.tw (L.-T.C.)

**Keywords:** fatherhood, depressed mood, postpartum, latent class growth analysis, Kids in Taiwan (KIT)

## Abstract

Parental psychological well-being is essential to the wellness of the family. However, longitudinal investigations into fathers’ postpartum depressed mood are limited. This study aimed to identify the typologies of depressed mood trajectories over the first year postpartum among Taiwanese fathers and to examine the factors associated with such typologies. We retrieved data from a nationwide longitudinal study on child development and care in Taiwan. A total of 396 fathers, who completed at least one of the three interviews when their children were 3, 6, and 12 months old between 2016 and 2017, were included in this analysis. Conditional latent class growth analysis was conducted to identify the classifications of the fathers’ depressed mood trajectories in the first year postpartum and to estimate the effects of covariates on individuals’ membership of a trajectory class. Three classes of depressed mood trajectories were identified. The high increasing group consisted of 11% of the participants; the moderate increasing and the low decreasing groups consisted of 28% and 61% of the participants, respectively. Financial stress was associated with the fathers’ likelihood of being in the high increasing group compared with their likelihood of being in the low decreasing group (OR = 2.28, CI = 1.16–4.47). The result may be related to the difference in gender roles and social expectations.

## 1. Introduction

Fathers and mothers respond and adjust to life-course changes in different ways. A two-way panel study showed that after the birth of a child, regardless of the first birth or a higher-order birth, the time that mothers spent on housework increased, while the time that fathers spent on housework remained relatively unchanged [1]. During the postpartum period, the fathers play a crucial role in supporting the mothers and taking care of the newborns. Mothers who perceive a lack of support from the spouse are more susceptible to feeling depressed after the birth of the child [2,3]. Although maternal postpartum depressive symptoms are extensively discussed in the literature, the fathers’ emotional changes during the postpartum period are also an important mental health issue. A recent meta-analysis showed that the prevalence of paternal depression from pregnancy to postpartum was 8.4% (95% CI, 7.2–9.6%), and the number varied among countries, with 12.5% for North American countries and 7.8% for Asian countries [4]. Overall, men’s depressive symptom scores had a significant increase, especially during the early stage of fatherhood, according to a population-based study [5].

Fathers’ psychological well-being affects the relationship with their spouses, as well as the relationship with their children. Fathers’ depressive symptoms are also linked to their children’s emotional and behavioral problems, especially to the conduct problems of the sons [6]. Nevertheless, compared to the literature on maternal depressed mood and symptoms, studies on fathers’ postpartum depressed mood are still limited. There are also individual differences in the progression of depressed mood and symptoms from pregnancy through postpartum observed in longitudinal studies on paternal depression. According to a recent Finnish study, there were three types of trajectory of depressive symptoms for fathers from pregnancy to 24 months postpartum, and the fathers with the high trajectory type experienced elevated depressed mood throughout the first year postpartum [7]. Having this information allows researchers and practitioners to identify the subgroup of individuals who are more susceptible to depression over time. 

Fathers suffer from depression during the postpartum period for a variety of reasons. Being first-time fathers [8,9], lower educational attainment and economic status [8,9], low perceived support [9,10], and marital dissatisfaction [9,10] are common factors that affect fathers’ depressed mood and symptoms. Being a parent is a different experience for fathers and mothers [11]. Researchers continue to underscore the significance of gender in understanding parental stress and psychological well-being [12]. For instance, Pollmann-Schult [13] found that financial costs of parenthood have a stronger negative effect on men’s life satisfaction, while women’s life satisfaction is often affected by time costs. Social norms embedded in each culture define an individual’s role in society and influence an individual’s interaction in various social contexts. An integrative study showed that demographic characteristics such as financial status, family relationships, and cultural background all contributed to the change of the psychological health among Chinese fathers during the transition to fatherhood [14].

Parents in different countries may perceive the burden and stress caused by the cultural norms and expectations toward parenthood. Gendered parenthood norms contribute to the differences in psychological well-being among fathers and mothers in different societies [15]. For instance, a study conducted in Jordan suggested that first-time fathers’ postpartum experiences are influenced by cultural beliefs of masculinity that inhibit their involvement in caring for their spouses and babies, as well as their expression of psychological distress [16]. In today’s society, fathers are still expected to be the economic resource of their families even though many mothers have entered the labor force [17,18]. In Taiwan, most of the families are dual-income families, and yet the cultural norm still emphasizes the traditional role of the fathers as the main economic resource and the mothers as the main caregivers of the children [19]. Therefore, postpartum depressed mood trajectories and predictors may be related to the gender norm for Taiwanese fathers due to the cultural context. 

In the current literature, most studies on postpartum depressed mood and symptoms focus on women. Moreover, research on paternal depressive symptoms from a longitudinal perspective is still limited, especially in non-Western counties. Several statistical programs have been developed to understand developmental patterns using longitudinal data. One of the techniques is called latent class growth analysis (LCGA). In the LCGA, a latent trajectory class variable is estimated to represent between-person differences in terms of developmental patterns [20]. Instead of assuming all subjects develop or progress in the same pattern, LCGA identifies the different patterns of the trajectory among the subjects [20]. For more heterogeneous subjects, LCGA is more suitable than conventional growth modeling [21]. Since previous research suggested multiple types of trajectories of depressive symptoms among Finnish fathers [7], the first aim of this study was to identify the classification of depressed mood trajectories among Taiwanese fathers over the first year postpartum using longitudinal panel data. The second aim of this study was to examine the factors influencing the patterns of the trajectories among the participants. We hypothesized that multiple classes of depressed mood would be identified by performing the latent class growth analysis. Furthermore, we hypothesized that the factors, such as being first-time fathers, education and economic status, perceived support, financial stress, and marital satisfaction would be associated with fathers’ depressed mood trajectory profiles.

## 2. Materials and Methods

### 2.1. Participants

This study utilized data from the nationwide longitudinal project, Kids in Taiwan: National Longitudinal Study of Child Development and Care (KIT) [22,23,24]. The aims of this project included developing a dataset of child development in Taiwan and investigating the long-term effects of the family and childcare environments on child development. The KIT project adapted the stratified two-stage probability-proportional-to-size sampling method and recruited two representative samples of children who were three months old and three years old in 2016. The parents or the main caregivers of the children gave informed consent if they agreed to participate in the project. For the three-month-old sample, the project staff interviewed the parents or the main caregivers and followed up with the family when their children were 3, 6, 12, 18, and 24 months old and annually afterward. To estimate the trajectories of fathers’ postpartum depressed mood and factors that affected the typologies, the present analysis only analyzed data from 396 married fathers who completed at least one of the parent-reported questionnaires at the target child’s 3-month-old (wave 1), 6-month-old (wave 2), and 12-month-old (wave 3) interviews.

The baseline demographic characteristics of the fathers in this study are reported in Table 1. About 52.3% of the participants were first-time fathers. For more than half of the fathers (57.3%), household income was in the middle and higher levels, and more than half of the fathers (62.1%) had a bachelor’s degree or higher.

The age of the responding fathers in the first wave ranged from 19 to 65 years old (M = 35.99, SD = 6.09). The correlation analysis found that age was not related to depressed mood score at all three waves (wave 1: r = −0.043, *p* = 0.398; wave 2: r= −0.044, *p* = 0.388; wave 3: r = −0.043, *p* = 0.399). In addition, current literature suggests that age is not one of the influencing factors for fathers’ postpartum depression [9], but being a first-time father at a young age is [8]. We decided to include the status of being a first-time father instead of the current age as one of the covariates.

### 2.2. Instruments

Depressed mood was measured by asking the fathers, “have you felt depressed in the past three months?” The fathers answered the question with a 4-point scale (1: never, 2: rarely, 3: sometimes, and 4: often). The proposed covariates included the father’s educational level, monthly household income, being a first-time father, perceived support, financial stress, and marital satisfaction. The perceived support was measured by asking the fathers, “is there enough help at home?” The perceived financial stress was measured by asking the fathers, “do you feel that your current financial situation is sufficient to pay for living expenses?” The marital satisfaction was measured by asking the fathers, “are you satisfied with your marriage?” and “do you work as a team with your wife to raise the child?” The perceived support, financial stress, and marital satisfaction were measured using a 4-point scale, ranging from (1) strongly agree to (4) strongly disagree. All covariates were measured at wave one. Because all constructs were measured with a single question, the reliability test was not conducted. 

### 2.3. Procedure

During each interview, a trained interviewer from the KIT project contacted the participant and obtained the participant’s consent. Then, a face-to-face interview with the participant was carried out. The research framework and the data collection of the KIT project were approved by the research ethics committee of National Taiwan University (No. 201408ES007) and by the research ethics committee of National Taiwan Normal University (No. 201707HS003).

### 2.4. Data Analysis

All analyses were conducted using Mplus 8.4, and the maximum likelihood estimator with robust standard errors was used to estimate the model [25]. For estimation, 500 initial stage random sets of starting values were used, and 70 final stage optimizations were carried out to reach convergence. The scores of the fathers’ depressed mood from the three interviews were analyzed using the latent class growth analysis (LCGA) to identify the typology of the trajectories of the postpartum depressed mood. For the subjects who had the missing value on any of the time points, the Mplus program used full information available in model estimation. First, an unconditional model was estimated. The model that produced the smallest Bayesian information criteria (BIC), smallest adjusted BIC, largest entropy, significant Lo–Mendell–Rubin adjusted likelihood ratio test (LMR-LRT) and bootstrapped likelihood ratio test (BLRT), and adequate sample sizes in each class were considered the best-fitted model [21,26]. 

Then, the covariates, including the father’s sociodemographic information, were added into the model estimation after the appropriate number of classes had been identified. A father’s trajectory in a conditional model was estimated by taking into account his probability of belonging to an individual group, the class-specific means, and covariance structures, as well as the father’s measures of the covariates [26]. Because there were 27 participants who had missing value in one of the covariates (24 missing in monthly household income, one in perceived support, and two in perceived working as a team with spouse), multiple imputations were applied for handling missing data in covariates. Despite the fact that a conditional model with covariates does not necessarily produce the same membership classifications [27], a covariate-based membership identification is recommended to avoid misclassifications [20]. Two parameters, i.e., intercept and slope, were estimated for each class. 

## 3. Results

### 3.1. The Depressed Mood at the Three Interviews

Table 2 showed the depressed mood frequency reported by the fathers at the three interviews. At the three months after the birth of the child, 10.1% of the fathers reported feeling depressed sometimes or often. The percentage decreased to 8.6% at the interview six months after birth and returned to 10.8% at the interview 12 months after birth. Overall, the average scores of the depressed mood at each wave did not vary much throughout the three interviews.

### 3.2. Classification of the Fathers’ Depressed Mood Trajectories from 3 to 12 Months Postpartum

**Figure 1:**
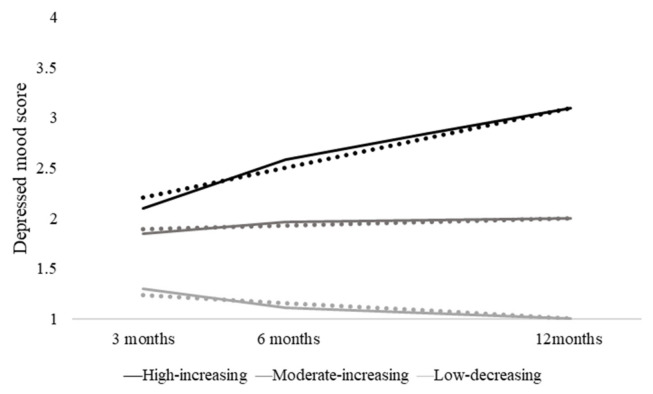
Estimated and actual mean trajectories of depressed mood among fathers. For each class, a solid line indicating the actual mean trajectory and a dotted line indicating the estimated mean trajectory are presented.

To identify the latent class of the fathers’ depressed mood trajectories, we performed the unconditional LCGAs and examined the models with two, three, and four classes. According to the fit statistics shown in Table 3, the BIC scores, as well as the adjusted BIC scores, decreased when the number of classes increased by 1. The three-class model produced the highest entropy score. Although the LMRLRT was not significant, the BLRT was significant for models with two, three, or four classes. In addition, one class in the four-class model consisted of only 4% of the sample, which did not exceed the cut-off ratio of 5% of the participants. Based on the fit statistics and the parsimony and interpretability of the models [20], a three-class model was selected. Figure 1 illustrates the depressed mood scores of the fathers and the three types of trajectories in the first year after the birth of the child.

### 3.3. Predictors of the Latent Class of the Fathers’ Postpartum Depressed Mood Trajectories

Based on the result of the unconditional modeling, a three-class conditional model with the covariates was estimated. The covariates included the father’s educational level, monthly household income, being first-time father, perceived support, financial stress, and marital satisfaction. At the baseline, the average score reported by these fathers on perceiving enough help at home, feeling financially sufficient, being satisfied with the marriage, and perceiving support from spouses were 2.06 (SD = 0.78), 1.96 (SD = 0.59), 1.51 (SD = 0.52), and 1.68 (SD = 0.57), respectively. Table 4 showed the proportion of each class and the parameter estimates of the latent classes. In total, 11% (n = 43) of the participants belonged to the first class (high increasing group), the trajectory of which displayed a high initial score and steadily increasing pattern over time. The second class (moderate increasing group), the trajectory of which displayed a moderate initial score and moderately increasing pattern over time, comprised 28% (n = 110) of the participants. The largest class (low decreasing group) with 61% (n = 243) of the participants displayed a low initial score and moderately decreasing pattern over time. The estimates of the intercept and slope parameters for all three classes were all statistically significant. 

As shown in Table 5, we did not find significant differences between the effects of the predictors on the moderate increasing group versus the low decreasing group. However, among educational level, monthly household income, first-time father (yes/no), perceived support, financial stress, and marital satisfaction, only financial stress was significantly associated, with 95% level of confidence, with the fathers’ likelihood of being in the high increasing group compared with their likelihood of being in the low decreasing group (OR = 2.28, 95% CI = 1.16–4.47). The fathers who perceived that their current financial status was not sufficient to pay for their living expenses were more likely to report a higher depressed mood score at the 3-month postpartum and their depressed mood escalated between the 3-month postpartum to the 12-month postpartum. In addition, the *p*-value of “not enough help at home” was 0.057, which was close to the cut-off level. The fathers who perceived insufficient help from home had a marginally higher chance to belong to the high increasing group.

## 4. Discussion

In the current study, Taiwanese fathers’ depressed mood trajectories over the first year following the births of their children were examined. About two-thirds of the participants were in the class with the lowest initial score and a moderately decreasing trajectory, a finding similar to that of Kiviruusu et al. [7]. However, unlike Kiviruusu et al. [7], which reported that the “high” profile among fathers had a moderate peak at 8 months postpartum, we found that the group with the highest initial scores reported an increasing pattern over time at least to 12 months postpartum. Our findings indicated that there were individual differences in the progression of depressed mood associated with fatherhood. The slopes for the two classes with lower initial scores were relatively modest, indicating that their depressed mood was somewhat stable. While most fathers rarely felt depressed when their children were born, some reported more depressed than their peers, and their depressed mood increased over time. The heterogeneity in fathers’ depressed mood trajectories is similar to the results found in mothers. According to several review articles [28,29,30], most studies on mothers’ depressed mood from pregnancy to postpartum identified at least three distinctive trajectories. While stability seems to be one frequently reported characteristic of these trajectories, mothers who felt depressed earlier during the postpartum period were more likely to develop chronic depression later on. We found that, among the fathers in this study, 10.8% felt more depressed at 3-month postpartum compared to the others, and their condition deteriorated from 3 to 12 months postpartum. Previous studies focused mostly on mothers, but our research provided findings showing that a subgroup of fathers also experience depressive symptoms during the postpartum period. It is important to track the progression of depressed mood after childbirth among fathers in order to identify those who are at risk of chronic depression. 

There was a correlation between financial stress and the typologies of depressed mood trajectories among fathers. According to the family stress model, economic hardship may cause economic pressure in the family, which leads to parental psychological distress [31]. In addition to the actual income and socioeconomic status, the perception of financial distress and financial satisfaction also have a stronger impact on a person’s psychological well-being. [32,33]. The results of our study showed that after controlling for the actual family income, the subjective evaluations of financial stress significantly predicted the fathers’ depressed mood trajectories. Hoebel et al. [34] suggested that subjective social status mediates the effect of objective social status on depressive symptoms in adults. In our study, fathers’ subjective perception of financial stress might fully explain the association between actual income and their depressed mood trajectories. Further investigation is warranted to examine the possible mediation effect of subjective financial stress. 

This study found that the net effects of marital satisfaction on fathers’ depressed mood trajectories were not significant as hypothesized, and the net effects of perceived support were not significant at the 95% confidence level, either. Although perceived support and marital satisfaction have been seen as strong predictors of maternal depressive symptoms [2,10], few studies examined the effect of marital satisfaction and perceived support on fathers’ depressed mood while controlling the effect of financial stress. Previous studies suggested that paternal depression and maternal depression are correlated, and the correlation can partially be explained by marital quality [35,36]. More dyadic studies of paternal and maternal depressed moods are needed to understand if marital satisfaction is only related to paternal depressed mood when maternal depression is present in the relationship. Nevertheless, the *p*-value of perceived support was close to 0.05. The small sample size may affect the power of the statistical result. More research with larger sample size is warranted to examine the association of perceived support and fathers’ postpartum depressed mood trajectories.

In Taiwan, traditional housework division and gender roles, through which fathers are the main breadwinners and mothers are the main caregivers in the families, are still prominent [37]. Lu [19] suggested that in the societies, such as Taiwan, that emphasize men’s responsibilities toward providing the family with financial security, money-related worries become the reason for men’s unhappiness. In our study, the only predictor of the patterns of the depressed mood trajectory for the fathers was the subjective judgment that their current financial situation was not sufficient to pay for the family living expenses. A study tracing the socio-economic panel data in Germany from 1984 to 2015 found that the weakening of gendered parenthood norms has contributed to overall life satisfaction in fathers and mothers [15]. Gender norms continue to play an essential role between parenthood and men’s and women’s psychological well-being. Besides socio-cultural explanations of fathers’ postpartum depressed mood, evolutionary psychology also provides a perspective to understand the trajectories. In Yu et al.’s study [38], motherhood had a stronger effect on mothers’ well-being compared to the effect of fatherhood. Moreover, paternal uncertainty further reduced the well-being of fathers. Further research may investigate the interaction between the socio-cultural perspective and evolutionary psychology perspective on parenting. 

Several strengths were highlighted by this study. First, this study used longitudinal data to examine the fathers’ depressed mood trajectories. This study was able to identify the individual differences in the trajectories of depressed mood over the first year after childbirth, especially for fathers in non-Western cultures. Moreover, this study examined how the change patterns of fathers’ depressed mood were associated with distinct factors, and we examined these in light of gender norms. Nonetheless, there are several limitations to the study. First, our data, which were retrieved from a child-focused survey, were secondary in nature; all variables, including the predictors and outcome measures, were collected through self-reporting. Because of the nature of the dataset, the small sample size may also reduce the power of this analysis. Furthermore, we used one question to measure parental depressed mood; although this approach captured the construct of interest, it is certainly less sensitive compared to a questionnaire with more items. Moreover, the fathers’ mood level before the childbirth was not available. The possibility of pre-existing depressive thoughts of the fathers in the high increasing group cannot be ruled out.

The present study was based on analysis of the data from fathers of the target child, who were available to answer the interviews in the original study; as a result, generalizing the outcome to fathers who don’t play a substantial role in child caring should be treated with caution. 

## 5. Conclusions

This study identified three classes of depressed mood trajectories among Taiwanese fathers over the first year postpartum. Moreover, this study found that perceived financial stress was associated with the fathers’ likelihood of showing the trajectory with a high initial score and steadily increasing pattern over time. Psychosocial resources and coping strategies are being introduced for new mothers during perinatal care [39]; however, fathers’ emotional needs and stress coping strategies are less discussed. Routine screening and social awareness should be implemented for fathers as well [40].

Garfield [41] proposed that the health care systems should be aware of the mental health issue of the father from the prenatal stage to the postnatal period. Supporting the father’s mental needs is supporting the healthy growth of the whole family. By exploring men’s experiences toward parenthood, we can gain a deeper understanding of this important moment in men’s life. An individual’s adjustment to this transitional period is influenced by gender roles and social expectations. To help the fathers to cope with the pressure that accompanies childbirth, it is important to understand the social and cultural factors associated with fathers’ psychological well-being. Most importantly, promoting awareness toward fathers’ psychological well-being is vital. Interventional programs that focus on improving paternal psychological well-being can benefit all family members.

## Figures and Tables

**Table 1:** Descriptive characteristics of the participants at the baseline interview (n = 396).

	n	(%)
First-time parent		
no	189	(47.7)
yes	207	(52.3)
Monthly household income		
low (USD <1000)	19	(4.8)
middle to low (USD ≥1000 and <2000)	126	(31.8)
middle (USD ≥2000 and <2667)	71	(17.9)
middle to high (USD ≥2667 and <3334)	83	(21.0)
high (USD ≥3334)	73	(18.4)
missing	24	(6.1)
Education level		
junior high school or lower	18	(4.6)
senior high school	84	(21.2)
vocational school	48	(12.1)
university	179	(45.2)
graduate school	67	(16.9)
missing	0	(0.0)

**Table 2:** Depressed mood of the fathers at three waves (n = 396).

	First Wave 3 Months After Birth	Second Wave 6 Months After Birth	Third Wave 12 Months After Birth
	n (%)		
1: never	231 (58.3)	233 (58.8)	243 (61.4)
2: rarely	124 (31.3)	128 (32.3)	110 (27.8)
3: sometimes	36 (9.1)	31 (7.8)	39 (9.8)
4: often	4 (1.0)	3 (0.8)	4 (1.0)
missing	1 (0.3)	1 (0.3)	0 (0.0)
	Mean (SD)		
Depressed mood scores	1.53 (0.70)	1.50 (0.67)	1.51 (0.71)

**Table 3:** Fit statistics of LCGA on fathers’ trajectories of depressed mood.

Classes	Bic	Abic	Entropy	LMRLRT *p*-Value	BLRT *p*-Value	Sample Size of Each Class (%)			
2	2050.09	2024.70	0.947	0.0009	0.0000	141 (36%)	255 (64%)		
3	1247.95	1213.05	1.000	0.56	0.0000	243 (61%)	110 (28%)	43 (11%)	
4	1240.91	1196.49	0.961	0.38	0.0000	43 (11%)	226 (57%)	110 (28%)	17 (4%)

Note: LMRLRT = Lo–Mendell–Rubin adjusted likelihood ratio test; BLRT = bootstrapped likelihood ratio test.

**Table 4:** Parameter estimates for each trajectory in the final model with covariates (n = 396).

	n (%)	Intercept		Slope	
		Estimate (SE)	*p*	Estimate (SE)	*p*
High increasing group	43 (11%)	2.19 (0.12)	0.000	0.30 (0.05)	0.000
Moderate increasing group	110 (28%)	1.88 (0.05)	0.000	0.04 (0.02)	0.000
Low decreasing group	243 (61%)	1.23 (0.03)	0.000	−0.08 (0.01)	0.000

**Table 5:** Logistic regression for predictors of trajectory class membership.

	Moderate Increasing (Ref. = Low Decreasing)		High Increasing (Ref. = Low Decreasing)	
	Estimate (SE)	OR (95% CI)	Estimate (SE)	OR (95% CI)
Education level	0.22 (0.12)	1.24 (0.99–1.56)	0.07 (0.16)	1.07 (0.79–1.46)
Household income	−0.01 (0.03)	1.00 (0.94–1.06)	0.01 (0.05)	1.01 (0.92–1.12)
First-time parent	−0.06 (0.24)	0.94 (0.59–1.50)	0.12 (0.36)	1.13 (0.56–2.28)
Not enough help at home	−0.01 (0.16)	0.99 (0.72–1.35)	0.41 (0.22) *	1.51 (0.99–2.30)
Not financially sufficient	0.09 (0.23)	1.09 (0.69–1.72)	0.82 (0.34) **	2.28 (1.16–4.47)
Unsatisfied about marriage	0.46 (0.29)	1.58 (0.89–2.79)	0.34 (0.38)	1.41 (0.67–2.94)
Spouse not a teammate	0.33 (0.28)	1.39 (0.80–2.43)	0.02 (0.31)	1.02 (0.55–1.88)

* *p*-value = 0.057; ** *p*-value = 0.016.

## Data Availability

The data presented in this study are available in Center for Survey Research, Research Center for Humanities and Social Sciences, Academia Sinica, Taipei, Taiwan at https://doi.org/10.6141/TW-SRDA-D00180-1, https://doi.org/10.6141/TW-SRDA-D00181-1, and https://doi.org/10.6141/TW-SRDA-D00214-2 (accessed on 20 December 2021).
